# Low intestinal parasite prevalence in Finnish pet dogs and cats

**DOI:** 10.1186/s13028-024-00776-4

**Published:** 2024-09-23

**Authors:** Pia Rapp, Eeva-Maria Williamson, Riina Uski, Inka Savikoski, Annika Pynnönen, Veera Gindonis, Antti Sukura, Anu Näreaho

**Affiliations:** 1 Department of Biosciences, Faculty of Veterinary Medicine, University of Helsinki, Helsinki, Finland; 2 Environmental Health Services, City of Porvoo, Porvoo, Finland

**Keywords:** Baermann, Canine, *Eucoleus*, Feline, Flotation, *Toxocara*

## Abstract

**Background:**

This study updates the knowledge of current canine and feline endoparasitic prevalence in Finland. The previous studies reported intestinal worm prevalence of 5.9% in dogs and 7.1% in cats. We also determined the anthelmintic regime and background data of dogs and cats concerning *Toxocara* spp. infection. Altogether 664 canine and 379 feline (including 46 shelter cats’) fecal samples from over six-month-old animals were examined with quantitative Mini-FLOTAC method using zinc sulfate with a specific gravity of 1.35. Of these samples, 396 canine and 89 feline samples were analyzed using the Baermann method for nematode larvae. A fenbendazole efficacy study was conducted with 12 animals positive for *Toxocara* spp.

**Results:**

Endoparasites were found in the feces of 3.5% of dogs, 3.6% of pet cats, and 41.3% of shelter cats. The most common findings in dogs were strongylid (1.7%) and *Toxocara canis* (0.9%) eggs. Trematode (0.4%), *Eucoleus* spp. (0.3%), taeniid (0.2%), and *Trichuris vulpis* (0.2%) eggs, and *Cystoisospora* spp. oocysts (0.2%) were also detected. One dog (0.2%) was positive for *Crenosoma vulpis* based on the Baermann method. *Toxocara cati* (3.3%), taeniid (0.6%), and trematode (0.3%) eggs were found in pet cats’ samples. The findings in shelter cat samples were *T. cati* (34.8%), *Eucoleus* spp. (13.0%), *Cystoisospora* spp. oocysts (10.9%), taeniids (8.7%), and *Toxoplasma gondii*/*Hammondia hammondii* oocysts (2.2%). Fenbendazole efficacy was adequate in all treated animals, except one cat. The background data revealed 31.2% of dogs being dewormed less than once a year or never. Under twelve-month-old dogs and dogs that were dewormed twice a year were most likely to be *T. canis*- infected. Shelter cats, male cats, mixed-breed cats, cats that were dewormed two to four times a year, and cats with a history of parasitic infections were most likely to be *T. cati* infected.

**Conclusions:**

The prevalence of pet canine and feline intestinal parasites in Finland is low, particularly the *Toxocara* spp. prevalence. In free-roaming cats *Eucoleus* spp. is surprisingly prevalent. The parasite control strategies reported do not follow the ESCCAP guidelines. Typically, owners deworm their pets only once a year or less frequently.

## Background

The pet dog population in Finland increased during the COVID-19- pandemic from 700,000 to over 800,000 individuals [1]. Cats are also popular pets; there were approximately 982,000 cats in Finland in 2021 [2]. Almost one-third of all Finnish households have at least one dog or one cat as a pet [3].

*Toxocara canis* in dogs and *Toxocara cati* in cats are globally significant intestinal worms in these species. Prevalence varies markedly between studied countries [4–16]. Based on questionnaire surveys, parasites are a common concern of pet owners [11, 16, 17]. However, the awareness of their zoonotic potential was only 24% among 206 respondents in Belgium and the Netherlands [16], 35% among 536 respondents in Portugal [17], and 49% among 185 respondents in Italy [11]. Endoparasites rarely cause visible symptoms for adult dogs and cats, but they can produce significant health issues for puppies, kittens, and immunocompromised individuals. Toxocariosis can also cause significant health issues in humans, especially small children [18]. Globally, human *Toxocara* spp. prevalence has been estimated at 19% based on a meta-analysis covering 250 studies from 71 countries [19].

In past Finnish pet animal endoparasite epidemiological studies, samples from dogs were collected in 2002 [20] and from cats in 2009–2010 [21]. The total intestinal worm prevalences were 5.9% and 7.1%, respectively. The most common intestinal parasite found in these data was *T. canis* in dogs (3.1% prevalence; 95% CI 1.9–5.1%) and *T. cati* in cats (5.4% prevalence; 95% CI 3.5–7.9%). Since then, recommendations for parasite control protocol have become more risk-based or diagnosis-driven in Finland, following guidelines by the European Scientific Counsel Companion Animal Parasites (ESCCAP) [22]. Importing dogs to Finland has also increased since the previous study [23], as well as the popularity of raw pet food diets which have more potential to transmit parasite infections, than feeds that have been heat treated [24]. In a survey conducted on pet owners in the UK, United States, Canada, Australia, and New Zealand, the habit of using raw food in the pet’s diet has increased between 2008 and 2018 from 16 to 66% in dogs and from 10 to 53% in cats [25]. The Finnish Food Authority has noticed an increase in registered raw food manufacturers in Finland during the last years [26].

Lungworm prevalence in pets has not been studied previously in Finland. Clinically, *Crenosoma vulpis* is diagnosed in Finnish dogs annually [27], and a survey from Finnish veterinarians published in 2019 suggested that individual cases of *Aelurostrongylus vasorum* have also been diagnosed [28]. Europewide feline lungworm prevalence was reportedly 11% among cats with outdoor access [29]. This dataset did not contain any of the Scandinavian countries. In England, 2% of cats were positive for *A. abstrusus* [30]. Based on the author’s (AN) experience, the clinical diagnosis of feline lungworms is absent or very rare in Finland.

Anthelmintic resistance has not been reported in dogs or cats in Finland so far, neither has it been studied, except for a small pilot study on cats [21]. Globally, resistant dog parasites have been reported since the 1980s in the United States and Australia [31, 32].

In this study, the status of canine and feline endoparasites in Finland is updated, their risk factors analyzed, and the prevalence of lungworms compared to assessed clinical observations. This study did not focus on the protozoans *Cryptosporidium* spp., *Giardia* spp., or *Tritrichomonas foetus*.

## Methods

### Sample collection and questionnaire

Fecal samples from 664 to 379 over six-month-old Finnish dogs and cats, respectively, were collected between February 2022 and October 2023. Pet owners and veterinarians were informed about the study in a national Kennel Club magazine, on the website of the Faculty of Veterinary Medicine, University of Helsinki, and in various social media groups targeted at pet owners and veterinarians. Canine samples were also recruited from two dog shows in the summer of 2022. If a household had several pets, only two dogs and two cats were accepted in the study. Stray cat shelters were contacted directly via email. Shelters were asked to take part in this study with cats that had recently been caught outdoors and to submit samples from these individuals before routinely deworming them. All the animals in the study, excluding those of shelter origin, had at least one month since their last anthelmintic treatment. Deworming history was not known for the animals originating from shelters. Fresh fecal samples were mailed to the laboratory on the same day as collected. In the laboratory, the samples were stored at 4 °C until processing within a week from sample arrival. All samples that had no proper identification, and samples that were too small or contained mold (due to prolonged time in the mail) were excluded from the study.

Each pet owner filled out the questionnaire on a website. The form contained questions about the pet’s breed, age, history of traveling abroad, current residential area, type of feed (including raw fish, raw meat), coprophagy, geophagy, deworming regime and frequency, the type of outings, living with other dogs and/or cats, possible symptoms, visits to the veterinarian during the last month, and possible earlier parasitic diagnoses.

### Quantitative flotation method

The fecal samples were first macroscopically inspected for aberrations such as cestode proglottids. The samples were then quantitatively analyzed with Mini-FLOTAC (Unit of Parasitology and Parasitic Diseases Department of Veterinary Medicine and Animal Production University of Naples Federico II, Naples, Italy) with a slightly modified protocol. Briefly: 2 g of feces were weighed, and 38 mL of zinc sulfate (specific gravity 1.35) was added. Due to a brief shortage of zinc sulfate, magnesium sulfate (specific gravity 1.29) had to be used for 42 dog and 34 pet cat samples. After careful mixing of the sample and the flotation fluid in a plastic cup with a metallic spoon, the mixture was sieved through a metallic tea strainer, mixed, and immediately after mixing pipetted into the Mini-FLOTAC chambers. After incubation for 10 min, the key in the Mini-FLOTAC disc was turned and both chambers were microscopically examined with x 100 magnification for parasite eggs and oocysts. All eggs or oocysts found in both chambers were counted and multiplied by 10 to gain EPG values (eggs per gram of feces). The sensitivity of Mini-FLOTAC is reported by the manufacturer to be 10 EPGs (in magnesium sulfate) when a dilution of 1:20 is used [33], which was also verified earlier with trematode egg-inoculated samples.

### Fenbendazole efficacy study

The owners of *Toxocara* spp. positive animals were asked to enroll their pets in a fenbendazole efficacy study. If they agreed, a correct dose (according to the animal’s weight, rounded upwards if needed) of over-the-counter (OTC) fenbendazole tablets (Axilur^®^ 250 mg or 500 mg depending on weight; Intervet International B.V.) were sent to the owner to be given to the animal over three consecutive days *per os*, as instructed by the manufacturer. Two weeks after the last medication day, the owner sent a new fecal sample of their animal for a fecal egg count using the Mini-FLOTAC method. Any problems with the medication, vomiting or diarrhea after administration was to be reported to the research group.

### Baermann

Samples with enough fecal material (at least 20 g) after the flotation method analysis were subjected to the Baermann method for detecting nematode larvae. To diminish the workload, five samples were pooled, i.e., 10 g of feces from each animal. The rest of each sample was left in a refrigerator until the analysis was finished. The samples were lightly mixed with a metallic spoon to allow better contact to the water phase for all samples and placed in a five-fold gauze cloth bag. The bag was then placed into a funnel with lukewarm water and incubated overnight (12–22 h). The next day, the sediment was either pipetted (system without a tap) or poured through a tap onto a Petri dish, with pre-drawn lines on the bottom to help the orientation during microscopy. The sediment liquid was examined with a stereomicroscope (Olympus model SZX-ILLB200, Tokyo, Japan) with x 20 to x 63 magnification. In case of a positive larval finding from the combined sample, individual 10 g samples of the positive batch were analyzed with Baermann to detect the positive animal(s). The identification of detected larvae was done based on larval morphology [34].

### Statistical analysis

95% confidence intervals (95% CI) were calculated for parasite prevalence, and for *Toxocara* spp. prevalence with different background variables. *Toxocara* spp. prevalence and their differences between background factors were examined with the chi-square test. Statistical analysis was performed with IBM^®^ SPSS^®^ 29.0.0.0 for Windows^®^. A *P*-value of under 0.05 was considered statistically significant.

## Results

### Dogs

A total of 664 dogs were included in the study. All dog fecal samples were examined with flotation, and 396 of these samples were also examined using the Baermann method. The questionnaire was completed for all participant dogs.

Mean age for participant dogs was 5.1 years, and median age was 4.2 years, with a range from 0.5 to 15 years. One-year-old dogs were the most represented group (14.5%), followed by two-year olds (13.1%) and over 10-year-olds (12.3%). Sixty percent of the dogs in this study were females (of which 67% were intact) and 40% were males (of which 72% were intact). Fifty-three dogs (8.0%) were mixed breeds, or their breed was not stated in the questionnaire. A total of 154 breeds were represented in the study population. Labrador Retriever was the most common breed (6.9%), followed by German Shepherd (3.5%), and Golden Retriever (3.2%).

Over half of the studied dogs reportedly spent time outdoors daily without a leash and 19% weekly. Under 1% (0.5%) did not have any free access outside. Raw meat or fish was consumed by 85.5% of the dogs as part of their diet. Soil and/or feces were eaten by 76.1% of the dogs. Traveling abroad was reported in the history of 26.4% of dogs in this study.

Routine administration was the most popular way of dispensing anthelmintic treatment: 72.9% of the dogs that were given anthelmintics were dewormed without prior fecal examinations. Twenty-eight (4.2%) dogs were reportedly not treated with antiparasitics nor were their fecal sample examined. Three dogs (0.5%) were left untreated based on a negative fecal exam result. Most of the studied dogs (32.8%) were treated with anthelmintics, or they had their fecal sample examined twice a year. The second most common frequency of anthelmintics or fecal sample examination was once a year (29.5%). 27% of dogs were treated with anthelmintics, or had their fecal sample examined less than once a year. Smaller groups of dogs were treated with anthelmintics, or had their fecal sample examined three to four times a year (5.0%) or over four times a year (1.5%). Some owners reported that their dogs (4.2%) were never treated with anthelmintics, or had their fecal sample examined. The most common anthelmintics administration or fecal examination frequency was twice a year (32.8%), with once yearly or less frequently being the next most common frequencies (29.5% and 27.0%, respectively). The time since a dog was last given anthelmintics was most often six months (31.6%) and over a year (29.8%), followed by three to six months (27.4%). Nearly one-third (30.1%) of the studied dogs had shown gastrointestinal and/or respiratory signs during the last four weeks and/or had been to a veterinarian in the last four weeks.

Fecal examination findings are shown in Table 1. Helminth eggs, protozoan oocysts, and/or nematode larvae were found in 3.5% of examined dog samples. The most common findings in fecal flotation were eggs of the Strongylida order. A dog that was positive for taeniid eggs was also positive for *T. canis* and a dog that had *Cystoisospora* spp. oocysts in its fecal sample also had *Eucoleus* spp. (syn. *Capillaria*) and *T. canis* eggs in the sample. The youngest dog that had intestinal parasites in the fecal sample was 10 months old and the oldest was 11 years old. Among the dogs positive for worm eggs, both rural and urban living areas were equally represented.

Among the samples tested with Baermann method, one dog (0.3%) was positive for larvae, and the larvae were identified as *C. vulpis* in the morphological analysis (based on larvae size and tail shape) [34].

**Table 1:** Parasite prevalence in studied dog population

Parasite	Positive (*n*)	Prevalence (%)	95% CI
Strongylida	11	1.7	0.8–2.9
*Toxocara canis*	6	0.9	0.2–2.0
Trematode	3	0.5	0.1–1.3
*Eucoleus* spp.	2	0.3	0.0–1.1
*Cystoisospora* spp.	1	0.2	0.0–0.8
*Trichuris vulpis*	1	0.2	0.0–0.8
Taeniid	1	0.2	0.0–0.8
Crenosoma vulpis*	1	0.3	0.0–0.8
All	23	3.5	2.2–5.2

Fecal flotation *n* = 664 dogs, Baermann method *n* = 396 dogs. The finding analyzed with Baermann method is marked with an asterisk (*)

The number of *T. canis* eggs in canine fecal samples ranged from 10 to 780 EPGs, with a median of 110 EPGs. The lowest and highest EPG values were found in under twelve-month-old dogs. In adult dogs, the range was from 40 to 110 EPGs. All six dogs positive for *T. canis* were of different breeds.

Four dogs took part in the fenbendazole efficacy study, and none of them had any *T. canis* eggs present in their fecal sample two weeks after medication.

Differences in *T. canis* prevalence between risk factors and their statistical significances are shown in Table 2. *Toxocara canis* infection was most common in dogs under twelve months old (prevalence 4.2%). The difference compared to over twelve-month-old dogs was statistically significant (*P* < 0.05). Other studied background factors were not significantly associated with *T. canis* prevalence (Table 2).

**Table 2:** The study population with number and percentage of T. canis-positive dogs

			T. canis-positive			
Variable		Total *n*	*n*	%	95% CI	*P*-value
Age group	6–11 months	71	3	4.2	0.9–11.9	0.019*
	12 months and older	593	3	0.5	0.1–1.5	
Sex	Female	399	3	0.8	0.2–2.2	0.687
	Male	265	3	1.1	0.2–3.3	
Traveling background	No	489	5	1.0	0.3–2.4	1.000
	Yes	175	1	0.6	0.0–3.1	
Multi-animal household	No	167	2	1.2	0.1–4.3	0.645
	Yes	497	4	0.8	0.2–2.0	
Grounds for anthelmintic	No anthelmintics	28	1	3.6	0.1–18.3	0.491
	Routinely	484	4	0.8	0.2–2.1	
	Fecal test	152	1	0.7	0.0–3.6	
Anthelmintic/fecal exam frequency	Never	28	0	0.0	0.0–1.23	0.288
	< once a year	179	0	0.0	0.0–2.0	
	Once a year	196	2	1.0	0.1–3.6	
	Twice a year	218	4	1.8	0.5–4.6	
	2–4 times a year	33	0	0.0	0.0–10.6	
	Over 4 times a year	10	0	0.0	0.0–30.8	
Previous parasitic infections	No	603	5	0.8	0.3–1.9	0.440
	Yes	61	1	1.6	0.0–8.8	
Time since last anthelmintic	Not known	16	0	0.0	0.0–20.6	0.117
	1–3 months	58	0	0.0	0.0–6.2	
	3–6 months	182	4	2.2	0.6–5.5	
	Over 6 months	210	2	1.0	0.1–3.4	
	Over 12 months	198	0	0.0	0.0–1.8	
Off-leash	Daily	401	5	1.2	0.4–2.9	0.562
	Weekly	126	1	0.8	0.0–4.3	
	Monthly	43	0	0.0	0.0–8.2	
	Periodically	91	0	0.0	0.0–4.0	
	Never	3	0	0.0	0:0–70:8	
Eating raw food	No	68	2	2.9	0.4–10.2	0.116
	Yes	568	3	0.5	0.1–1.5	
	Not known	28	1	3.6	0.1–18.3	
Geo/coprophagia	No	159	0	0.0	0.0–2.3	0.344
	Yes	505	6	1.2	0.4–2.6	
Signs or veterinarian visit within the last month	No Yes	464 200	3 3	0.6 1.5	0.1–1.9 0.3–4.3	0.373

Percentage and total number of *Toxocara canis-*positive dogs in the studied population (*n* = 664) by variables with 95% confidence intervals (95% CI) and *P*-values describing the statistical differences. A statistically significant *P*-value is marked with an asterisk (*< 0.05)

### Cats

This study included 379 cats, 46 of which were stray cats recently taken to shelters. The questionnaire was completed for all pet cats and for one stray cat. All fecal samples were examined with flotation, and 89 samples were also examined with the Baermann method. All samples examined with the Baermann method were from pet cats.

The mean age of pet cats in this study was 6.7 years and the median was 5.8 years, with a range from 0.5 to 22 years. The one-year-old group (12.6%) was the largest represented age group, followed by one-year-olds (11.2%) and three-year-olds (9.0%). 52% of pet cats in this study were female (of which 34% were intact) and 48% were male (of which 19% were intact). Half of the pet cats were purebreds (*n* = 170), and 163 cats were mixed breeds, or their breeds were not stated in the questionnaire. The age or breed of the shelter cats were unknown. Eight of the shelter cats were reported to be female, one was spayed. Sixteen of the shelter cats were male, one of them was castrated. No gender data were available for 22 of the shelter cats.

**Fig. 1:**
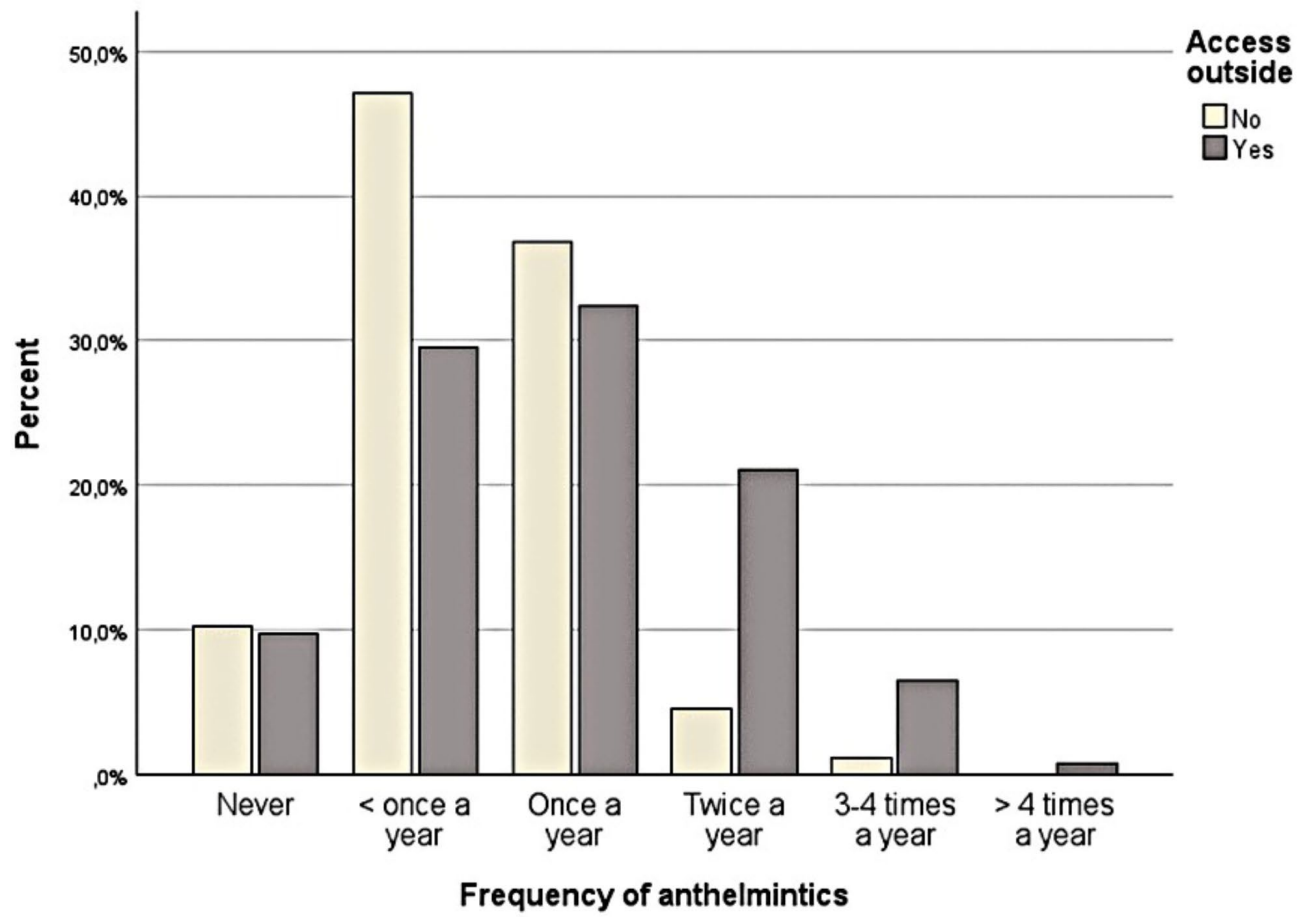
Frequency of deworming by outdoor access in the studied Finnish pet cat population (*n* = 334)

Over half (74.0%) of the studied pet cats had outdoor access, either freely, on a leash, or in a pen. Raw food (meat, fish, or prey animals) was consumed by 81.1% of the pet cats. Most of the studied pet cats had no history of traveling abroad (86.8%). Over half of the pet cats (56.0%) were treated with anthelmintics routinely without a fecal examination for parasites, and 11.4% of the pet cats had reportedly never been treated with anthelmintics. The most common frequency of anthelmintic treatment or fecal sample examination was less than once a year (34.1%). The next common frequencies for anthelmintics or fecal sample examination were once a year (33.5%) and twice a year (16.8%). Some cats were treated with anthelmintics, or had their fecal sample examined three to four times a year (5.1%) or over four times a year (0.6%). Almost 10% of cats (9.9%) were reported never to be treated with anthelmintics, or had their fecal sample examined. Almost half of the studied pet cats (41.6%) were last treated with anthelmintics over a year earlier. This was followed by cats that were dewormed three to six months before sampling (20.7%) and over six months before sampling (15.9%). For 10.2% of pet cats, it was unknown when deworming last took place. The deworming frequency for cats with or without outdoor access is presented in Fig. 1. Cats that had outdoor access were most often treated with anthelmintics once a year. Over one-third (35.9%) of the studied cats had shown gastrointestinal and/or respiratory signs or they had been to a veterinarian during the last four weeks.

The fecal examination findings are shown in Table 3 for pet cats and in Table 4 for shelter cats. Worm eggs and/or protozoan oocysts were found in 3.6% of pet cat samples. In shelter cats, 41.3% of the samples were positive for intestinal worm eggs and/or protozoan oocysts. *Toxocara cati* was the most common finding in both cat groups. Taeniid and trematode eggs were also found in pet cats. *Eucoleus* spp. eggs were found in 13.0% of shelter cat samples and in none of the pet cat samples. No nematode larvae were found in feline fecal samples examined with the Baermann method. The youngest pet cats infected were eight months and the oldest 10 years old.

**Table 3:** Parasite prevalence in studied pet cat population

Parasite	Positive (*n*)	Prevalence (%)	95% CI
*Toxocara cati*	11	3.3	1.7–5.8
Taeniid	2	0.6	0.1–2.1
Trematode	1	0.3	0.0–1.7
All	12	3.6	1.9–6.2

Samples were examined with fecal flotation (*n* = 333 pet cats) and Baermann method (*n* = 89 pet cats)

**Table 4:** Parasite prevalence in studied stray cat population

Parasite	Positive (*n*)	Prevalence (%)	95% CI
*Toxocara cati*	16	34.8	21.4–50.2
*Eucoleus* spp.	6	13.0	4.9–26.3
*Cystoisospora* spp.	5	10.9	3.6–23.6
Taeniid	4	8.7	2.4–20.8
*Toxoplasma/Hammondia*	1	2.2	0.1–11.5
All	19	41.3	27.0–56.8

Samples were examined with fecal flotation (*n* = 46 stray cats)

The number of *T. cati* eggs in the fecal samples ranged from 20 to 6,050 EPGs, with a median of 570 EPGs. The median for pet cats was 360 EPGs and for shelter cats 595 EPGs. The EPG value of under 12-month-old *T*. *cati-*positive pet cats (*n* = 3) ranged between 170 and 4,760 (median 360) and between 20 and 6,050 (median 460) in over twelve-month-old pet cats (*n* = 8).

Eight cats were included in the fenbendazole resistance study. One cat had an insufficient response to the treatment; the fecal *T. cati* egg count was 390 EPG after medication. This cat was treated for an additional three days with fenbendazole, and no *T. cati* eggs were found in its feces two weeks after the second treatment. A new fecal sample was examined again after two additional weeks, and no *T. cati* eggs were found in the feces.

Number and percentage of *T. cati* positive cats by different variables with 95% confidence intervals are shown in Table 5.

**Table 5:** The study population with number and percentage of T. cati-positive cats

			*T. cati-* positive			
Variable		Total *n*	n	%	95% CI	*P*-value
Age group	6–11 months	29	3	10.3	2.2–27.4	0.060
	12 months and older	305	8	2.6	1.1–5.1	
Sex	Female	182	8	4.4	1.9–8.5	0.041*
	Male	175	18	10.3	6.2–15.8	
Shelter background¹	Yes	46	16	34.8	21.4–50.2	< 0.001***
	No	333	11	3.3	1.7–5.8	
Traveling background	Yes	44	0	0.0	0.0–0.8	0.371
	No	290	11	3.8	1.9–6.7	
Multi-animal household	Yes	281	10	3.6	1.7–6.4	1.000
	No	53	1	1.9	0.0–1.0	
Anthelmintic regime	No anthelmintics	38	0	0.0	0.0–9.3	0.026*
	Routinely	187	10	5.3	2.6–9.6	
	Fecal test	109	1	0.9	0.0–5.0	
Anthelmintic/fecal exam frequency	Never	33	0	0.0	0.0–10.6	0.022*
	< once a year	114	2	1.8	0.2–6.2	
	Once a year	112	3	2.7	0.6–7.6	
	Twice a year	56	2	3.6	0.4–12.3	
	2–4 times a year	17	4	23.5	6.8–49.9	
	Over 4 times a year	2	0	0.0	0.0–84.2	
Previous parasitic infections	No	271	4	1.5	0.4–3.7	0.001***
	Yes	63	7	11.1	4.6–21.6	
Time since last anthelmintic	Not known	34	0	0.0	0.0–10.3	0.133
	1–3 months	39	3	7.7	1.6–20.9	
	3–6 months	69	4	5.8	1.6–14.2	
	Over 6 months	53	2	3.8	0.5–13.0	
	Over 12 months	139	2	1.4	0.2–5.1	
Outdoor access	No	87	3	3.4	0.7–9.7	1.000
	Yes	247	8	3.2	1.4–6.3	
Eating raw food	No	50	0	0.0	0.0–7.1	0.096
	Yes	271	11	4.1	2.0–7.1	
	Not known	13	0	0.0	0.0–24.7	
Geo/coprophagia	No	306	11	3.6	1.8–6.3	0.609
	Yes	28	0	0.0	0.0–12.3	
Signs or veterinarian visit within the last month	No	214	10	4.7	2.3–8.4	0.105
	Yes	120	1	0.8	0.0–4.6	
Breed	Mixed breed	163	10	6.1	3.0–11.0	0.005**
	Purebred	171	1	0.6	0.0–3.2	

Percentage and total number of *Toxocara cati-*positive cats by variables with 95% confidence intervals (95% CI) and *P*-values describing the statistical differences. Statistically significant *P*-values are marked with an asterisk (*< 0.05, **<0.01, *** <0.001). Shelter background (marked with ¹) was analyzed as a variable for all participant cats (*n* = 379). Other variables were calculated only for pet cats (*n* = 334)

## Discussion

This study shows a decreasing prevalence of intestinal parasites in Finnish dogs and pet cats compared to earlier reports. The prevalences established in this study were 3.5% in dogs and 3.6% in pet cats. *Toxocara* spp. prevalence was 0.9% in dogs and 3.3% in cats. The previous prevalence studies conducted on Finnish dogs and cats revealed endoparasitic prevalences of 5.9% and 7.1%, respectively [20, 21]. The most common finding in these studies was *Toxocara* spp. in both species (prevalence 3.1% in dogs and 5.4% in cats). The study published in 2006 [20], which included dogs of all ages, observed a prevalence of 12.1% (95% CI 9.5–15.2%) for dogs between 12 weeks and 12 months of age. This included eggs from the order Strongylida along with *T. canis* eggs. The previous study published on Finnish cats [21] also included cats of all ages, and they observed a *T. cati* prevalence of 6.7% (95% CI 3.0–12.9%) among cats under 12 months of age. One potential explanation for the lower total prevalence in the current study may be that we excluded animals under six months of age. Small puppies and kittens are commonly *Toxocara* spp. infected, and they are routinely given anthelmintics until four months of age. We therefore wanted to exclude this population from our dataset and set the six-month criterion for sampling. The age group of 6–12 months in this study had the highest *Toxocara* spp. prevalence (4.2% in dogs and 10.3% in cats). In dogs, only the age of less than 12 months was found to be a statistically significant risk factor (*p* = 0.019) for *T. canis* infection in this study, but the number of positive cases was low. Keeping the zoonotic potential of *Toxocara* spp. in mind, attention should be directed to animals of up to 12 months of age, and not only to small puppies and kittens, when making recommendations of deworming or diagnostics. A recent study of 303 Swedish adult dogs revealed an endoparasitic prevalence twice as high as in the current study: 7.9% [14]. Nearly half of the dogs (43%) in the Swedish study had their fecal samples collected over three days, which increases the sensitivity. Only dogs whose fecal samples were collected over three consecutive days, were included in the Baermann examination, and still no larval stages of nematodes were found [14]. Only a one-day sample was requested in the current study, as this study was not for diagnostic purposes. The Finnish canine endoparasitic prevalence is very low when compared to the Europewide fecal examination results in a study conducted on 2469 dogs in 12 countries: 7.6% of dogs were positive for nematodes, and 3.6% were infected by ascarids [35].

The dogs in this study represented different age groups, genders, and breeds, and they were recruited from various sources without exclusion or inclusion due to previous signs. The three most common dog breeds in this study were among the five most popular breeds in Finland in 2023 according to The Finnish Kennel Club in 2024 [36].

The variety of parasites in this study was somewhat small. Eggs of the order Strongylida were the most common finding in the dog samples (*n* = 11, prevalence 1.7%). *Uncinaria stenocephala* and *A. caninum* are globally common strongylids of dogs, but herbivores are also infected by strongylids, and dogs are keen to eat herbivore feces. In this study, the identification of strongylid egg species was not attempted. Seven of the strongylid findings were observed with concurrent presence of *Eimeria* spp. oocysts, which were identified based on their morphology. *Eimeria* spp. are coccidians of herbivores that are commonly found as pseudoparasites in the fecal samples of coprophagic dogs [37]. In this study, 14.6% of dogs had *Eimeria* spp. oocysts in their feces. This implies that strongylid eggs also very likely originated from herbivore feces consumed by the dog. This makes *Toxocara* spp. the most common pathogenic parasite finding in this study in both dogs and cats. *Uncinaria stenocephala* prevalence in Finnish dogs was 2.6% in the previous study [20], but all strongylid findings were included in this number. One dog was found positive for *T. vulpis* (prevalence 0.1%), and a low prevalence of this parasite is in line with the previous study (0.2%) [20]. *Cystoisospora* oocysts, trematode and taeniid eggs were observed in the current study but not in the previous one [20]. A flotation solution with higher specific gravity was mainly used because we were also interested in the presence of liver flukes in the dog and cat populations. Fluke eggs are heavy and may not float well in regularly used flotation solutions. This could be the reason why trematode eggs were not discovered in the earlier study. One of the three dogs positive for trematodes had morphologically identical trematode eggs to *Dicrocoelium dendriticum*, a common fluke of sheep and deer, for example. In a further examination, this dog was kept strictly on a leash for three consecutive days, and no trematode eggs were found in the fecal sample afterwards. This suggests that the previous result was due to coprophagy. Even though 46% of the pets in this study reportedly ate raw fish daily or occasionally, no *Diphyllobothrium latum* eggs were found in the fecal samples. In the last Finnish canine study, *D. latum* prevalence was 0.4% [20]. The general prevalence of *D. latum* in Finland has decreased along with the increased use of water closets and the consequent disruption of the life cycle [38].

Cats in this study had lower taeniid prevalence than in the previous study (1.7%) [21] despite a high-density flotation solution and a more sensitive method used. *Cystoisospora* spp. oocysts were not detected in this pet cat material compared to a prevalence of 0.7% in the study published in 2012 [21]. This is probably due to differences in the two study populations. However, one cat was found to be positive for trematodes, and this was a new finding compared to the previous study [21]. The fact that mainly zinc sulfate was used instead of magnesium sulfate (which was used in 2012) increases the likelihood of fluke egg flotation. The trematode-positive cat had no signs of illness but showed a significantly increased fasted bile acid level in serum. After treatment with praziquantel, the bile acid value resolved to normal, and no trematode eggs were found in the two following fecal samples.

Only one canine sample with nematode larvae was found using the Baermann method, suggesting that asymptomatic, non-diagnosed infections in the Finnish dog population are not common. Most of the fecal sample collection in dogs was conducted during winter and spring, but pet cats were included in the Baermann method part of the study in fall. *Crenosoma vulpis* infects dogs via snails, and usually the clinical cases start to appear after the prepatent period in fall. However, clinical *C. vulpis* cases in dogs have previously been diagnosed in Finland also in spring [27]. No previous prevalence reports compiled using the Baermann method exist for Finnish dogs or cats, but occasional cases of *C. vulpis* and rare cases of *A. vasorum* have been diagnosed from patient samples [27, 28]. Also, a few cases of intestinal *Strongyloides stercoralis*, which is also diagnosed with the Baermann method, have been found previously in Finnish dogs [39]. The decision to use pooled fecal samples of several individuals for Baermann screening was made to decrease the workload in the laboratory. A 10 g sample/animal was used and at least 10 g of each sample left for subsequent individual testing in case the combined sample was positive. In commercial laboratories, a 5 g sample is routinely used for Baermann analysis [40], which usually consists of feces from three different days. The authors are confident that the 10 g sample is quite representative, but, obviously, the intermittent shedding of larvae could be missed on low-shedding days.

Fenbendazole is the most commonly used/sold *per os* anthelmintic for dogs in Finland (Chairman Marttila, Eläinlääketeollisuus ry, Finnish Association of Veterinary Drug Industry, personal communication 2024) and was therefore selected for this efficacy study. It affects nematodes, certain cestodes, and *Giardia* spp [41]. , but only *Toxocara* spp. positive animals were included in this part of the study. A small pilot study has previously been published in Finland, with the FECRT of *T. cati-*positive cats (*n* = 9) treated with pyrantel, with no eggs detected in the post-medication sample [21]. In the current study, no conclusions were made about the possible *Toxocara* spp. resistance for fenbendazole due to the small number of positive samples. The owner of the cat that still had eggs in the post-medication sample reported no problems during medication, but since the tablets were not given by a veterinary professional, the possibility of unsuccessful administration must be kept in mind. However, the authors believe it is also important to occasionally follow the efficacy of anthelmintic treatments in small-animal samples, especially in kennels and catteries, not to be caught off-guard by anthelmintic resistance. With canine and feline samples, the egg count can be assumed to be zero after medication.

Gender of pet or traveling abroad did not seem to correlate with higher *T.* canis prevalence in dogs. Dogs entering Finland must have a mandatory *Echinococcus* medication given, and sometimes this can be combined with nematode drugs. Thereby a recent travel abroad may have lowered the parasite prevalence due to medication. Most of the *T. canis* positive dogs had unleashed outdoor access, but no statistically significant differences were observed between the groups. In this data, eating raw food did not increase the risk of *T. canis* infection in dogs, but on the contrary decreased it. A previous study covering 938 dogs in the Netherlands also found eating raw food to appear as a protective factor against *T. canis* infections [42]. Geo- and/or coprophagic dogs were more prone to *T. canis* infections in the current data, which was also found in the previous study published in 2016 [42]. *Toxocara* spp. eggs are common in soil since pooled prevalence in public areas globally has been shown to be 21% [43]. In children the habit of geophagy is known to be a risk factor for *Toxocara* spp. infections [44].

Over 30% of dogs were treated against worms less than once a year or never, even though the guidelines established by ESCCAP recommend either deworming or fecal examinations for parasites every six to 12 months, even for dogs and cats with a low risk for parasite infections [22]. However, this rarely treated group had no *T. canis* positive cases, which indicates that the individuals’ parasite risks are low, and that the owners know it. One-third of the dogs in this study were dewormed twice a year, whereas the previous Finnish study found the corresponding percentage to be nearly two-thirds [20]. In the same study, 1.7% of dogs were never dewormed during adulthood [20], whereas now this share of dogs was over twice as large (4.2%). Dogs that were treated with anthelmintics twice a year were more likely to be infected with *T. canis*, implying that the owners are aware of their dogs’ parasite risks, and therefore deworm them every six months. The habit of deworming dogs twice a year (after winter and after summer) could also be a remnant of the old recommendations that veterinarians issued.

The shelter cats and pet cats were analyzed separately due to their completely different living conditions and because the histories of the shelter cats were unknown. Cats from shelters were more likely to be infected, as was expected based on their predatory background before capture. Pet cats of less than twelve months of age were more likely to be infected with *T. cati*, which was also the case in the earlier feline prevalence study performed in Finland [21]. The prevalence in this age group of pet cats was higher in the current data (10.3%) than in the previous study on Finnish cats (8.1%) [21], even though the latter study also included under six-month-old kittens. Male cats were significantly more likely to be infected with *T. cati* than females were. Being a mixed breed was connected to a more probable *T*. cati infection, as was also the case in the earlier Finnish study [21]. This is probably due to their different lifestyle compared to purebred cats. Cats with outdoor access were as likely to be *T. cati* infected as cats with no outdoor access. This is different from the earlier Finnish study, where outdoor access was found to be an obvious risk factor for *T. cati* infection. Possibly, *T. cati*-positive cats that had no outdoor access in this study had contracted their infections prior to the beginning of their indoor lifestyle. In the current data, raw food was also connected to more *T. cati* infections, so some infections could have originated from feed. In the previous Finnish cat study [21], the same higher *T. cati* prevalence was found with raw food. Cats that were dewormed routinely and two to four times a year were most likely to be *T. cati*-positive. Possibly the owners of these cats treat them with anthelmintics frequently, being aware of their cats’ risk behavior, but as the risk of infection (eating prey) is constant, *T. cati* prevalence remains higher than in the other groups.

Shelter cats had high intestinal parasite prevalence, as was expected since cats contract *T. cati* and taeniid infections when eating small rodents as prey. In shelter cats, *Eucoleus* spp. were the second most common parasites in this data, with a surprisingly high prevalence (13.0%). All shelter cats positive for *Eucoleus* spp. were caught in the southeastern part of Finland. However, the majority of the examined shelter cats were from this area, so no geographical conclusions can be made. *Eucoleus* spp. were not found in the previous prevalence study [21], nor are they commonly diagnosed in veterinary clinics. In this study, the likelihood of finding *Eucoleus* spp. eggs in fecal examination could have been increased by the use of zinc sulfate of high specific gravity as a flotation solution. No attempt was made to identify the species, but the respiratory nematode *Eucoleus aerophila* (syn. *Capillaria aerophila*) and the intestinal nematode *Capillaria putorii* (syn. *Aonchotheca putorii*) have been reported in cats [9, 29, 45, 46]. There is also a report of *Eucoleus boehmi* (syn. *Capillaria boehmi*) eggs found in a cat’s fecal sample in Denmark [47]. *Eucoleus boehmi* can cause upper respiratory symptoms and typically infects canids [48]. Stray cats may eat earthworms as easy prey, and it is hypothesized that earthworms can act as paratenic or intermediate hosts of *Eucoleus* spp [49, 50]. Given the relatively high prevalence found in this study, more attention should be paid to feline *Eucoleus* spp. infections in Finland, especially with outdoor cats. For comparison, a study conducted on stray cats in Northern Germany, *Eucoleus* spp. prevalence was 5.0% [9].

It should be regarded that strongylid EPG values can somewhat decrease in zinc sulfate of high specific gravity as was used in this study [51]. However, when diagnosing *Toxocara* spp. infections, zinc sulfate with a specific gravity of 1.35 has been found to be the most efficient flotation solution [52]. It should also be noted that flotation methods are not very sensitive in the diagnostics of cyclophyllidean cestodes, as most of the eggs are within the proglottids. This makes the taeniid prevalence findings of 0.2% in dogs, 0.6% in pet cats, and 8.7% in shelter cats underestimates of the true prevalences. This should be kept in mind also when analyzing the samples for anthelmintic treatment needs; the possibility of cestode infection (due to eating intermediate hosts) should be based on information from the owner, and cestode medication should be administered in cases where risk behavior is observed.

## Conclusions

Intestinal parasite prevalence in dogs and cats in Finland is low. However, pets between 6 and 12 months are at risk to be infected especially with *Toxocara* spp. This should be kept in mind when planning recommendations for parasite control after the first few months of the pet’s life. Routine deworming of over 12 months apart, which was reported quite commonly, does not meet the recommendations by ESCCAP.

Cats that have been brought to a shelter have high endoparasite prevalence. *Eucoleus* spp. was surprisingly prevalent among them, which should be considered when planning their anthelmintic treatments.

The trematode findings in dogs and a cat in this study raise the question of whether fecal samples should be more often examined in a flotation solution of higher specific gravity especially in pets that have hepatic signs or liver enzyme changes of unknown cause.

The very low prevalence of respiratory nematodes in this study confirms that it is reasonable to keep examining fecal samples with the Baermann method only from symptomatic animals.

## Data Availability

All but identification data are available from the corresponding author upon request.

## Declarations

### Prior publication

The data have not been published previously.
